# Process- and outcome evaluation of an orientation programme for refugee health professionals

**DOI:** 10.1080/10872981.2020.1811543

**Published:** 2020-08-24

**Authors:** Sidra Khan-Gökkaya, Mike Mösko

**Affiliations:** Department of Medical Psychology, University Medical Center Hamburg-Eppendorf, Research Group on Migration and Psychosocial Health, Hamburg, Germany

**Keywords:** Refugees, health professionals, orientation programme, evaluation, labour market

## Abstract

Background

Refugee health professionals experience several barriers on their path to re-entering their original occupations in host countries. Several training programmes exist in order to address these barriers and enable a successful labour market integration.**Objective**: This study aims to evaluate a specific orientation programme for the labour market integration of refugee health professionals in Germany. The programme lasts three months and comprises three elements (German technical terminology language course, cross-cultural coaching and a job shadowing).**Design**: A mixed-methods design was implemented to evaluate the programme. To assess participants’ skills improvement and satisfaction, self-developed questionnaires were used. For in-depth-evaluation of individual experiences, qualitative interviews were conducted at four time points (pre, half-time, post, follow-up) with both programme participants and programme providers about their experiences.**Results**: Participants described impacts on their personal situation and improvement of their language, professional and formal skills. Some participants also described negative effects mostly due to unsupervised shadowing. Additional barriers, such as cost of travel were identified as challenges for participation.**Conclusion**: Training programmes may affect language skills, professional skills and formal resources. However, programme providers need to anticipate negative effects and introduce actions for preventing negative outcomes. Moreover, programmes should be designed to reduce work-related fear and anxiety amongst participants.

## Introduction

Due to the increasing numbers of refugees in Europe [1] their integration into labour markets has become a key challenge. Programmes for specific occupational groups have been implemented to address the labour market integration of refugees. As there is a shortage of health professionals in several European countries [2], occupational specific programmes have been implemented focusing on refugee health professionals. However, refugees belong to a particularly vulnerable group within the labour market of host countries [3] as their access to the labour market and to language courses often remains restricted [4]. Especially the access to jobs in the health care sector is described as challenging as health professions are highly regulated [5]. The recognition of their prior qualifications [6,7], a lack of proof of qualifications [8,9] and unfamiliarity with the health care system [10] pose barriers for successful integration. The experience of discrimination [11], the loss of their professional identity [12] and the loss of self-confidence [13] are additional psychological and structural barriers. Therefore, it is recommended by the European Parliament to implement tailored programmes focusing on on-the-job-training of refugees [14]. Depending on the target group and the aim of the programme, the design and curriculum of the programmes vary. Most programmes described in literature address international health professionals [15] or international medical graduates [16,17]. Findings from a systematic review on programmes for international medical graduates pose the assumption that programmes need to focus on increasing self-efficacy whilst reducing work-related stress and anxiety [16]. However, most of the programmes have in common that they lack methodological quality and transparency in terms of evaluation methods [15–18]. Thus, it remains unclear how effective programmes are in this context. This underlines the importance of focusing on the effectiveness and the outcomes of such programmes [15]. Thus, this study aims to systematically evaluate an orientation programme for refugee health professionals.

## Methods

### Programme description

The programme that is subject to investigation was funded by the European Social Fund for four years and implemented as a pilot-programme at the University Medical Center Hamburg-Eppendorf. The programme targeted refugee health professionals from all health professions who applied or are preparing to apply for the recognition of their qualification in Germany. As a precondition to participate in this programme, refugee health professionals must be registered in Hamburg. The programme was designed as an orientation programme enabling refugees to gain insights into their potential workplaces before entering the labour market and learn occupational specific German. Thus, the programme did not directly lead to a qualification that is recognised by the licensing bodies for recognition.

The programme lasted three months and comprised three major modules: (1) German technical terminology language course, (2) cross-cultural coaching and (3) job shadowing. Due to legal regulations, foreign health professionals without a work permit are not allowed to perform a hands-on internship. Thus, a hands-off approach was conducted where participants were only allowed to watch. For the job shadowing, participants were matched with a ward corresponding to their qualifications. They shadowed at their wards for three days a week. Additional two days were reserved for the German technical terminology language course and the cross-cultural coaching. The German technical terminology language course focused especially on medical communication situations and case studies. The German course was supported through blended learning using online and in-person components. An online learning platform was established which provided interactive video and audio exercises for participants. Through blended learning participants were enabled to widen their professional vocabulary and encouraged to use their language competencies. The (face-to-face) cross-cultural coaching focused on cross-cultural aspects of clinical work such as communication, interprofessional work, confidentiality, and relationship towards patients. Part of the cross-cultural coaching was also to strengthen participants in dealing with difficult situations such as experiencing racism, solving conflicts with team members and preparing for job interviews through role plays. Participants were neither paid for participation nor did they had to pay for participation except for travel costs and food expenses.

#### Aim of the study

The aim of this study was to conduct a mix-methods process- and outcome evaluation including a three-month follow-up of an orientation programme for refugee health professionals. The mixed-methods process of evaluation addressed four questions following the Kirkpatrick Training Evaluation [19] framework:

1. How satisfied were participants with the programme?
2. What programme aspects were challenging for participants and what programme aspects can be improved?
3. Did participants gain knowledge through the programme, and if yes, in what areas?
4. What other outcomes could be observed through the programme?

### Study design

**Figure 1.**
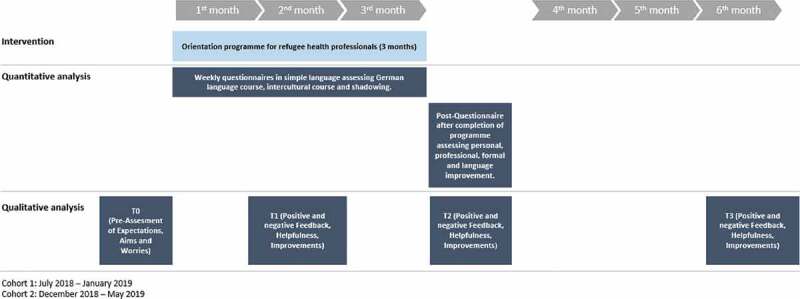
Evaluation design of the orientation programme for refugee health professionals.

The mixed-method study design comprised a qualitative case series concept [20] and two quantitative questionnaires. All participants were included in the quantitative evaluation. For in-depth evaluation and a broader understanding of the impact that the programme had made on the participant’s situation a qualitative case study concept [20] was chosen. Figure 1 refers to the evaluation design of this study.

### Instruments

#### Quantitative process data acquisition

Due to a lack of previously validated instruments for this specific context, a questionnaire was developed. The questionnaire was developed according to the heterogeneous composition of the group with regard to language competencies. The weekly questionnaires were designed in plain language to assess the three parts of the programme (German technical terminology course, cross-cultural coaching, job shadowing). The questionnaire assesses the satisfaction and relevance of each part of the programme corresponding to the first level of evaluation according to Kirkpatrick (reaction) with two items on a 4-point Likert scale and open-response questions.

#### Quantitative outcome data acquisition

In order to assess the second level of Kirkpatrick’s training evaluation model (learning), participants completed a self-developed post-questionnaire at the end of the project. The questionnaire was based on three identified outcome-levels from a previous review on qualification programmes for immigrant health professionals [18] and was developed with additional experts in migration research. These outcome-levels refer to (1) improvement of language skills such as learning new vocabulary or improving ones communication skills in clinical work, (2) improvement of personal and professional skills, such as being more confident, gaining knowledge about the health care system or being less afraid to speak German, (3) the improvement of formal resources/career prospects such as getting in contact with other health professionals, being encouraged to work, improving their CVs and finding internship or job placements. The questionnaire consisted of 16 items on a 5-point Likert scale. In order to include all participants and minimise language barriers, the German questionnaire was translated into Arabic and Farsi as most participants were fluent in either one of these languages.

#### Qualitative data acquisition

Interviews were conducted with 24 participants^1^ from two cohorts (N = 29) of the programme at four times: one interview before the programme started, one after halftime, one at the end of the programme and one three months^2^ after finishing the programme. Two teachers and one practical instructor from two cohorts were also interviewed at three times (pre, halftime, and post). The guideline specifically asked for assessment of the whole programme, every single part of the programme, positive and negative feedback of the programme as well as suggestions for improvement. For the qualitative, in-depth evaluation participants needed to have sufficient language competencies and a high chance of participating in the programme for the entire period. Participants received two consent forms: one for their participation in the study and one for their consent for audio recording. The consent form and the study information were also explained orally prior to the interview. For every interview the same guideline was used. The interviews lasted between four minutes to 38 minutes with a median range of 18 minutes. Demographic data was retrieved after the first interview. The interviews were conducted by Sidra Khan-Gökkaya, a female, person of colour, PhD student at the Department of Medical Psychology with several years of experience in conducting qualitative studies in the field of migration research and two research assistants, both female and psychology students.

### Data analysis

The questionnaires were analysed using SPSS 18 and descriptive data. The interviews were transcribed verbatim by a professional agency and analysed by the first author using qualitative content analysis according to Mayring [21] by means of a computer-based coding programme (MAXQDA, version 10). Within the process of analysing data deductive categories were derived from the interview guide. However, due to the explorative approach of the study more inductive categories were derived from the material itself. Code memos for the categories were created and included a description of the code and typical quotes. For the purpose of quality assurance two research assistants coded a quarter of all interviews independently. Uncertainties in coding were discussed. They led to the creation of some new sub codes.

Qualitative and quantitative results were presented and discussed in an interdisciplinary research colloquium with experts from the research group to ensure comprehensibility and utility.

## Results

Table 1 provides a summary description of the study sample. Table 2 presents an overview of identified codes and sub-codes used to organise findings. Results are divided into five sections based on order of data collection.

**Table 1.** Sample characteristics.

	Cohort 1	Cohort 2	Cohort 3	Total
Target group	Participants	Participants	Staff	
Sample size	N = 17	N = 12	N = 3	N = 32
Sex	Male (n = 12) Female (n = 5)	Male (n = 6) Female (n = 6)	Female (n = 3)	
Country of origin	Syria (n = 12), Algeria (n = 2), Kongo, Iran, Moldova	Syria (n = 9) Afghanistan (n = 2) Iraq, Lybia	Germany (n = 3)	
Profession	Physician (n = 14), Psychologist (n = 2), Medical technical assistant	Physician (n = 7) Physiotherapist, Dentist, Pharmacist, Medical technical assistant, Nurse	German teacher, cross-cultural trainer, practical instructor

**Table 2.** Overview of identified codes and sub-codes used to organise findings.

Time Point	Theme	Codes	Subcodes
T0	Qualitative Pre-Programme Assessment	Pre-Programme Assessment	Referral to the project Aims Expectations and Worries
Process Evaluation			
T1/T2	Qualitative Process evaluation	Overall programme organisation	Availability of staff Composition of programme Shadowing opportunity
T1/T2		German technical terminology course	Practise oriented teaching Positive relationship towards teacher
T1/T2		Coaching	Reflection about cultural aspects Reflection about own experiences
T1/T2		Challenges with German technical terminology course and coaching	Differing language competencies Spatial equipment Timing Focus on medical topics only
T1/T2		Job Shadowing	Insight into work routine Encouragement from colleagues Support from medical students
T1/T2		Challenges with Job Shadowing	No person responsible Distanced relationship towards colleagues Job shadowing placements Technical issues
T1/T2		Improvements	Job Shadowing Overall organisation of programme Additional offers
T1/T2		Psychosocial Stress factors	Acquiring language competencies Recognition of qualification and work Personal and social situation Travel expenses
T1/T2		Staff Experience	Language and occupational differences within participants Suggestions for improvement
T1/T2	Quantitative Process Evaluation		Results of weekly questionnaires Assessment of Satisfaction and Importance
Outcome Evaluation			
T2/T3	Qualitative Outcome evaluation	Impact and Improvement of skills	Personal situation Language skills Professional skills Formal resources None or Negative impacts (T3/T4) Outcomes observed by staff
T2	Quantitative Outcome Evaluation		Results of Post-Questionnaire Assessment of improvement of language, professional and formal skills (see Table 3)

**Table 3.** Results post-questionnaire **^a.^**

	n	Strongly dis-agree	Dis-agree	Partially agree	Agree		Strongly agree	Mean	SD
LANGUAGE SKILLS:									
My German has improved through the project	24	-	-	46%	29%		25%	3.79	0.83
I learned new technical language	25	-	-	24%	44%		32%	4.08	0.76
I’m less afraid of speaking German while working	25	-	-	20%	36%		44%	4.24	0.78
I can communicate better while working	23	4%	9%	13%	35%		39%	3.96	1.15
PROFESSIONAL SKILLS:									
I’m more self-confident while working than before.	23	4%	9%	13%	35%		39%	3.96	1.15
I feel better prepared to work in Germany	23	-	-	22%	52%		26%	4.04	0.71
I know more about the health care system	24	8%	-	8%	33%		50%	4.17	1.17
I know more about the working culture in German hospitals	23	4%	4%	13%	35%		44%	4.09	1.08
I got a good insight into the German hospitals	23	4%	-	17%	44%		35%	4.04	0.98
I gained new medical knowledge	23	-	-	35%	35%		30%	3.96	0.83
FORMAL SKILLS									
I was able to build professional networks because of the programme	23	17%	17%	22%	39%	4%		2.96	1.22
I was encouraged to work in my job again	23	4%	4%	9%	30%	52%		4.22	1.09
I will find a job easier	18	6%	6%	39%	28%	22%		3.56	1.10
I found a job	20	80%	10%	5%	5%	-		1.35	0.81
The programme helped me with my professional career here in Germany/Hamburg	20	15%	10%	30%	30%	15%		3.18	1.27
I feel better prepared for the language and knowledge assessment	23	9%	-	35%	30%		26%	3.65	1.15

^a^Values are rounded.

### Qualitative pre-programme assessment

Participants described that they were referred to the programme through employment agencies, other integration-projects or other refugee health professionals. They participated in the programme in order to refresh their knowledge to improve their professional performance and increase their follow-up chances for internships or job placements. Participants were worried about the process especially regarding the shadowing as they feared they would not be accepted or supported at the ward. Furthermore, they were afraid their German would not suffice for participating in the programme.

### Qualitative process evaluation

#### Overall programme organisation

Participants were generally satisfied with the programme. They positively commented on the availability of the project staff and fast support in cases of questions related to the recognition of their qualification or other personal and social issues. They also described that the project gave them an opportunity to shadow which is not easy to get. Participants also emphasised the division between German technical terminology language course, cross-cultural coaching and job shadowing at the end of the programme:

I think that is good [the division of the programme]. When we notice something or if we don’t understand something during the job shadowing we can bring that up later in the course. And explain and discuss what was wrong about it or what was right about it (P35).^3^

#### German technical terminology course

Participants highlighted the relevance of the German technical terminology course as they all wanted to improve their language skills. In this context, they benefitted from a practice-oriented teaching in which patient-consultations were simulated. They highlighted their positive relationship towards teachers and expressed that receiving feedback from teachers was very helpful. They also positively highlighted interaction between the group and exchange of ideas.

#### Cross-cultural coaching

Participants described that the topics of the cross-cultural coaching were relevant for them. They did not only gain information about the health care system but also reflected on cross-cultural aspects in Germany:

I have been in Germany for two years and I did not have proper contact to German culture. And knowing what a taboo is here, what is not right, what one should not do. That is good to know before having contact to patients (P21).

They also considered the cross-cultural part as not only helpful for their professional skills but for their overall situation in Germany:

It [the cross-cultural coaching] has got to do with everyday life. […] She discussed a challenging topic with us- racism. Something we experience every day- [we talked about] how you can find a solution with regard to this topic or a way out of these situations. […] not only in the medical area, generally (P104).

#### Challenges with German technical terminology course and coaching

Participants criticised the overall composition of the group as they all had differing language competencies:

What I don’t like is that we are not on the same level in the course. […] Everyone has another level of competencies. Sometimes this is very boring for some people. Sometimes they have material that is for all people. But for those people who know the material it is boring. And sometimes it is difficult for other people to understand this (P21).

Participants also criticised the spatial equipment as room changes for German class and cross-cultural coaching happened frequently and they were not informed about these changes. They were also dissatisfied with the coaching especially with regard to timings. Some participants felt uncomfortable as the classes took place Friday afternoon and this timing colluded with Friday prayers for Muslims. Participants described participation in Friday prayers as very important for their well-being and stress-reduction. Thus, they always had to balance between courses/coaching and prayers.

Participants also felt misplaced and reported not benefitting from the programme as much as their colleagues who were physicians:

Sometimes I had the feeling of being in the wrong place. Because I knew it would not help me in my future life. […] Because in my area, for example the laboratory, for me it is important to speak about blood, about microscopes, about bacteria, about a lot of things. But the content in the teaching course was important for physicians or nurses (P52).

#### Job shadowing

Participants described ambivalent experiences with the job shadowing. They highlighted that they gained insights into daily work routine in Germany and familiarised with these routines.

I was expecting to get an idea how this works. What are the tasks of a junior physician? What does a junior physician do? How does he talk to senior physicians? These little things, I had no idea about. And know through job shadowing I have a better idea about that (P35).

They highlighted the importance of interpersonal relationships with team members and supervisors. Positive feedback from colleagues was perceived as encouraging:

The physicians [at the ward] are very friendly and they encourage me to find work. They always say: ‘Yes, you can do this. Apply to all hospitals in Hamburg’. And I have heard that several times. It encouraged me because I did not have the feeling of being prepared for work. (P21).

They benefitted from students who were completing their medical/practical year at the ward:

I personally benefitted a lot from them and I had a very good relationships towards students. For example, I told them I needed a physician’s letter to learn. And every week twice or thrice they helped me to write the letter (P73).

#### Challenges with job shadowing

A major critique point was that no person felt responsible for inducting them throughout their job shadowing:

The problem at the ward is, I don’t have any work. No one helps me finding orientation. For example, I come every morning at six o’clock and I try to do something- but no on helps me. That is my problem. [I feel] Like a stranger at the ward. That is why I think the shadowing is boring. (P26).

They also pointed out that physicians did not have any time to talk to them and they had a distanced relationship towards team members:

They [team members] are under time pressure. […] And especially the culture. I don’t know how to behave best or how to get closer to them. […] Never has someone said to me ‘What do you want? What do you want to learn?’ It is this distance every time (P29).

As a consequence of not being allowed to work or not being taken care of, participants got bored.

They also criticised the overall organisation of the job shadowing as some of them were not even assigned to a job shadowing, were assigned to it very late or were assigned to a ward that did not match their qualification. They also criticised some technical issues such as not having access to the computer system, not having keys for the physicians’ room and not knowing where to change their clothes.

#### Suggestions for improvements

Participants proposed organising job shadowing placements that correspond with individual qualifications for all participants before the beginning of the programme. They also proposed to integrate some additional offers such as lectures from experienced colleagues and professors, discussing patients’ cases and establishing voluntary mentors they could work with. With regard to improvements of the overall programme, they proposed a review of the teaching courses and coaching timings, the composition of the group and access to technical and spatial resources such as to patient files and to changing rooms. They also proposed offering specialised workshops for different professions and for participants with higher and lower language competencies. Regarding the appropriate length of the programme, participants gave mixed feedback.

#### Psychosocial stress factors

Throughout interviews, participants mentioned other aspects that seemed to influence their performance and their well-being. Acquiring sufficient language competencies was perceived as very difficult:

The language barrier was my biggest fear. […] My confidence decreased a little bit. Because of the language. I think I was a good student at my university […] And when I worked I was good at my work. But then after coming to Germany the language was a big fear (P69).

Furthermore, they were worried about their professional future prospects and how to cope with all the barriers connected to re-entering their professions in Germany:

I hope I get this paper [from the licensing bodies] and my work permit [permit to work as an intern] and then I can do the internship and learn for the knowledge examination. We say ‘you can’t carry two melons with one hand’. I am carrying two melons in one hand. But I need to hold on to them (P85).

Participants also mentioned their personal or social situation which was influenced by the circumstances of their flight or their status as refugees in Germany: ‘As a refugee in German it is hard to find employment. I am afraid of racism or discrimination, that makes me worry’ (P58).

Another participant described his worries about his family and the role of the project:

I always think about my parents. Because they are in trouble. They need help and I’m not there. […] And that is why I always think about it. […] When I work, especially in the laboratory, then my head is busy with working and learning. […] If I get a job shadowing placement, then I’m in it (P66).

However, this participant could not be matched to a job shadowing placement.

Financial barriers such as travel expenses also influenced participation as employment agencies did not cover travel expenses:

I like it [the job shadowing]. But that is a problem for me. Because it is halftime [halftime of the programme]. And if I want to catch up on the missed time [participant was assigned to a job shadowing 6 weeks after the start of the programme] then I would have to add one or two months. And if that is the case there are problems with travel expenses. Because the ticket is 160€ per month (P27).

#### Staff experience

Facilitators and teachers experienced similar challenges as participants. It was a challenge for them to include all students despite their language and professional heterogeneity:

We had two psychologists. […]. Thematically, very less [of the used material] was suitable for them. But those communication aspects, speaking German, acting in role plays. I think they could pick up on that (P30).

They also experienced a hardship in finding job shadowing placements:

We have a few [job shadowing placements] now we can always rely on. But, all these different specialties, if participants have different occupations, dentists or dermatologists, it is always very difficult. (P70).

They observed that as a consequence of the described challenges in the context of job shadowing, participants were demotivated. They also noticed that not being able to actually do something was frustrating for participants. They proposed to establish a mentoring programme so that someone at the ward would feel responsible for them. They also suggested introducing specific workshops such as the use of the computer system and creating welcome documents that mentors or ward staff can hand out to participants on their first day.

### Quantitative process evaluation

#### German technical terminology course (quantitative results)

On average, German classes were attended by 21 out of 30^4^ participants in both cohorts (range = 18–25), which corresponds to an average attendance of 70%.^5^ On average, participants were generally ‘satisfied’^6^ with the German technical terminology course (mean = 3.37, SD = 0.56, range = 2–4) and rated the topics of each session as ‘important’ (mean = 3.38, SD = 0.60, range = 2–4).

#### Cross-cultural coaching (quantitative results)

With regard to the cross-cultural coaching, an average of 18 out of 29 participants in both cohorts attended individual sessions, which corresponds to an average attendance of 62.1% (range = 10–23).^7^ On average, participants were ‘very satisfied’ (mean = 3.58, SD = 0.51, range = 2–4) with the cross-cultural coaching. In addition, the topics of each session were rated on average as ‘very important’ (mean = 3.55, SD = 0.53, range = 2–4).

### Qualitative outcome evaluation

Participants described initial outcomes after halftime of the programme. The programme affected their personal situation and it contributed to the improvement of their language and professional skills as well as to their formal resources.

#### Impact on personal situation

Participants described programme participation as a reasonable alternative to doing nothing:

I can’t stay at home. [Improve] linguistically and medically. Collect information. I can’t stay home because that makes me sick because I have stayed home for a long time. I don’t have a workplace. This is why I also want to participate in job shadowing’s or internships (P20).

#### Improvement of language skills

Participants described that their language competencies have improved. They highlighted that they have learned new health-related vocabulary and became used to standardised medical communication situations such as talking to patients and colleagues:

I noticed the change. Before the job shadowing I didn’t talk too much German. But after the beginning of the programme until now my German has improved a lot. Maybe I don’t talk very well now, but it’s not like before. […] Also, when I talk to patients now, it happens very rarely that I don’t understand their answers. It was not like that in the past (P72).

Participants related their success in their language examinations to programme participation:

My first month with the job shadowing was neither very well, but I was happy, because I learned German. It helped very well, to speak German. It helped to pass the language examination. It [the job shadowing] played a big role [in passing the examination] (P85).

#### Improvement of professional skills

Participants described that their personal and professional skills also improved. By not being a stranger they felt more secure:

When I apply for a job, when I go to work, I am not the stupid person standing around and not knowing what to do. No, I can show that I know the system. That can build trust (P21).

Their self-confidence was also increased by speaking and listening to German:

If you hear the language, that helps. […] I talked to my teacher and my colleagues and that helped me a lot. I had more self-confidence (P72).

They describe that they felt more encouraged and more secure than before:

The project encouraged me and now I can begin to work. Maybe. In the past I had a different idea how work in hospital is organised. […] And the German language was very complicated for me. And I was afraid to work. But, now with the job shadowing- at the beginning it was very hard. But, now it is easier. I had three job interviews during the three months (P40).

After three months participants also described that they lost their fear and were less reluctant to speak German:

I was afraid. But now I can just work. Just talk to patients and say everything without fear. […] And it [the programme] gave me some orientation how to work in hospitals or deal [with others]. […] I was afraid of talking German. And here I tried. Physicians and patients did not have time […] I did not have that many chances [to talk to them]. But, I talked to nurses and several times to patients. And that was very good (50).

#### Improvement of formal resources

Participants described that participating in the programme improved their CVs and was helpful for their career:

I think the work I found now, it was easier to find it through the project. Because, they saw it in my CV. You were at the UKE [abbreviation for University Medical Center], you did an internship there. You speak very well, you have experience, you know what you are doing. And in that way it helped me (46).

One participant was also able to gain an internship placement after the job shadowing which she needed for recognition of their qualification. Moreover, at the time of follow-up participants had stayed in contact, established learning groups or exchanged important information regarding their recognition and job interviews which increased their formal resources.

#### None or negative impacts

After the end of the programme, there were also participants who reported none or negative impacts. Depending on their previous knowledge, participants described that no impact was observed in their language skills or their knowledge about certain topics. Negative effects were mostly reported due to their unsupervised job shadowing: Some of them reported that they felt superfluously as the ‘sixth finger’ at the ward and it was not possible to proceed ‘happily and strongly’ (91). Lack of supervision during job shadowing or not having a job shadowing placement at all led to frustration and disappointment of participants:

I am disappointed. Unfortunately I did not had practical experience [a job shadowing placement] in this hospital to talk about it. That was a big disappointment for me (52).

One participant was negatively affected in his self-confidence by the shadowing experience:

I had a lot of expectations from this project. It is in the University Medical Center and it is a big University Medical Center. You can learn a lot but then the reality was totally different. I was a little bit disappointed. I need to say that in English. A little bit of my confidence, was a little bit down. […] Because the people there didn’t know about the project. They have no idea what the project is and who the new people are (104).

Likewise, there were participants who did not stay in contact with other participants. Thus, no sustainable networks were built.

#### Outcomes observed by staff

The teachers and facilitator agreed that all participants could improve their language skills and were better prepared for working in health care. Furthermore, they highlighted the role of the networks that were established between participants. However, the success of the programme was also connected to the personal situation of the participants:

Several of them who have other big problems. Seven people in one and a half room with corresponding issues in the background, with children. You can be happy when someone like that can concentrate on the course. If someone is threatened with deportation, it is also difficult. And of course all this is difficult to predict (83).

### Quantitative outcome evaluation

#### Improvement of language, professional and formal skills (quantitative results)

In addition to the weekly questionnaires, a self-developed post questionnaire was handed out to the participants from two cohorts (N = 29) after the end of the programme. Three participants did not fill out the questionnaire. Reasons for non-participation are unclear. Participants’ ratings in the category of language skills improvement were between 3.79^8^ (SD = 0.76) and 4.24 (SD = 1.15). Participants highly agreed to almost all the items (except for Item ‘I found a job’). One person strongly disagreed to communicating better while working.

The item ‘I am less afraid of speaking German while working’ received the highest agreement. Improvement of personal and professional skills were rated by the participants between 3.96 (SD = 0.71) and 4.17 (SD = 1.17). They all agreed that they felt better prepared to work in Germany. However, one participant disagreed with being more self-confident than before and three participants disagreed with knowing more about the health care system and the working culture in Germany. All of them agreed that they have learned new medical knowledge. The improvements of formal skills were rated between 1.35 (SD = 0.81) and 4.22 (SD = 1.27) although in this category there was a wide range of responses. However, most of the participants strongly agreed that they were encouraged to work in their jobs again.

## Discussion

This study aimed to evaluate an orientation programme for refugee health professionals by implementing a mixed-methods design. Due to the increased number of refugees and a lack of qualified health professionals in European countries, there is a need to implement orientation and qualification programmes. At the same time, it is necessary to systematically evaluate these programmes in order to make sure that they are effective and sustainable. However, research indicates that several challenges occur when conducting quantitative studies with refugees. For example, linguistic and cultural specificities within the target group need to be considered [22]. Moreover, refugees may be reluctant to participate as they fear negative consequences due to participation or non-participation [23]. Corresponding with literature, we also experienced several challenges in conducting the evaluation in this study.

### Methodological challenges

The first challenge refers to the language barriers within the target group. The composition of participants was very heterogeneous with regard to their German language competencies and their native languages. These language barriers may have influenced the interview process. Moreover, they made it challenging to use validated instruments as it could not be assumed that all participants would understand German questionnaires. Furthermore, there were no specific instruments such as a self-efficacy scale or a sense-of-belonging scale to evaluate within this specific context. With regard to these barriers, the self-development of questionnaires corresponding to the design and the aims of the programme seemed reasonable. Nevertheless, by conducting a mixed-methods study and evaluating qualitatively, a differentiated picture of the effects of the programme became obvious. Thus, it can be assumed that conducting a mixed-methods study design may on the one hand be extensive and challenging, but on the other hand very informative especially when favouring an explorative approach. For research purposes it is also necessary to critically reflect the usefulness of qualitative and quantitative methods in the context of programme evaluation and to consider the challenges with target groups when developing appropriate instruments. Aside from the restrictions of the self-assessment [24] and a limited translation quality [25] the quantitative evaluation was helpful as it identified specific aspects that have not been detected by the qualitative approach alone.

### Programme design

#### Programme elements

Participants were generally satisfied with the design of the programme. They positively pointed out the division between German technical terminology course, cross-cultural coaching and shadowing. As some participants had difficulties finding shadowing placements on their own, they could benefit from the job shadowing opportunity within the programme. Qualitative results suggest that participants who were satisfied with their job shadowing (placement) were generally satisfied with the programme. On the contrary, participants who did not receive a shadowing placement or were not supervised at their placements were disappointed. According to the programme providers, it was very hard to find job shadowing placements. Participants also described that colleagues at the ward were very busy and did not seem to have time. This may be due to the precarious staffing situation in German hospitals [26] and not specific to the programme site. However, as the programme site is a University Medical Centre offering students and interns regularly placements, there seems to be potential for optimisation.

As language skills are essential for participating in the labour market, it is not surprising that all participants were satisfied with the German technical terminology language course and rated its topics as important. They pointed out the benefits of a practise-oriented learning as specific situations and cases at wards could be picked out. Participants were also satisfied with the cross-cultural coaching and rated its topics as very important as quantitative results suggest. Thus, it can be assumed that addressing cross-cultural aspects is very important for a successful labour market integration. This is consistent with qualitative findings. Some participants described that they were insecure about cultural standards, as cultural standards are often implicit [27]. Moreover, results reveal that cross-cultural coaching was not only helpful in the context of labour market integration but also for their overall situation in Germany as they learned strategies to deal with challenging situations.

#### Group composition

Participants in this study benefitted from each other and liked exchanging ideas in the group. Previous studies suggest that teaching different professions together is fruitful in terms of acculturation [16]. However, some of them were not satisfied with the group composition. Participants had different language competencies which made the German technical terminology course and cross-cultural coaching sometimes difficult for participants with lower competencies and boring for participants with higher competencies. Additionally, participants who were not physicians felt misplaced and discouraged as topics in the German technical terminology course and cross-cultural coaching seemed to focus on this profession. There seems to be a challenge in designing programmes for all health professionals and addressing relevant issues for all professions. This may also be the reason why there are so few programmes aimed at all professional groups [18]. Differentiating the curriculum and offering specialised workshops corresponding to competencies and professions as proposed by the participants may be difficult due to limited resources and unequal distribution of the professions. However, integrating group work into the existing design of the course could be one opportunity to give participants time to specifically deal with issues that are relevant for their profession.

#### Time aspects

Some participants mentioned the timing of the classes as challenging as it coincided with the timing of Friday prayer. They described that participation in Friday prayers was very important for their well-being and stress-reduction. This is consistent with previous findings that religion can be a coping strategy for refugees [28] and a ‘source of emotional and cognitive support’ [29]. It underlines the important role of support systems in this context and matches observations from staff that some participants were burdened by the circumstances that led them flee their home country and the experiences as a refugee.

Participants gave mixed feedback on the appropriate length of the programme. Some participants who were satisfied with their job shadowing wished for a longer duration. Others who were bored at their wards suggested shortening the length of the programme. As the programme was designed as an orientation programme not leading to a recognised qualification a short duration seems reasonable.

### Programme effectiveness

#### Impact on personal situation

Considering the restrictive integration policies in many European countries, it is not surprising that refugees see the programme as a welcoming diversion. It is also consistent with previous findings that restrictions in employment negatively affect the situation of refugees resulting in deprofessionalisation [30] and slowing down the economic integration of refugees [31]. Moreover, the longer refugees stay out of the professional field, in an unskilled profession or on welfare, the harder it gets to re-enter their professions [32]. Thus, supporting structures and occupational specific programmes should be offered more often instead of restricting access to supporting structures for refugees. In this context, it is also important to reduce additional barriers for participation in programmes. Results revealed that for example travel expenses posed a barrier for some participants. It is consistent with previous findings that financial hardships can pose a barrier to obtaining registration as well as to retraining [33]. Thus, employment agencies and programme providers should consider covering for travel expenses in order to prevent non-participation due to financial barriers.

#### Improvement of language skills

For participating in the labour market it is essential to have good language skills. Qualitative and quantitative results prove that most of the participants could improve their language skills in this three-month orientation programme. Some participants who already had very good language competencies described that they did not benefit from the programme at all. A heterogeneous group composition with regard to language competencies, foreign language competencies and learning habits is a common problem in the context of language acquisition [34]. However, integrating partner and group work or introducing team teaching may be helpful [34]. Improvement in language skills and communication can also be associated with improvement of self-confidence and a reduction of fear. The highest rated item in the questionnaire was related to being less afraid of speaking German.

#### Improvement of professional skills

Throughout the study it became obvious that fear was a manifest feeling of participants. Participants described that they were afraid that their language competencies would not suffice. Moreover, they were afraid of discrimination and rejection at the ward. Additionally, they were also afraid of their chances to enter their jobs. Thus, it can be concluded that a lot of fears are connected with workplace participation. These findings indicate that programmes should focus more on reducing work-related fear and anxiety. In the programme, especially the cross-cultural coaching aimed to strengthen participants in dealing with difficult situations and preparing them for their path to a successful labour market integration. Qualitative results show that they were strengthened for example in dealing with racism and that they perceived the coaching not only as helpful for their professional career but for their general situation in the new country. Results also indicate that the cross-cultural coaching was perceived as a space for reflecting their experiences and finding their own strategies in dealing with challenging situations. Moreover, quantitative results prove that the programme had an effect on participants’ self-confidence and that they were encouraged. One participant explicitly mentioned the positive feedback from his colleagues as encouraging. Considering these effects it can be concluded that programmes for refugee health professionals should focus even more on empowering them. This could be done by emphasising their qualifications such as their multilingualism instead of only focusing on the lack of language competencies. Moreover, it is necessary to point out the qualifications and experiences that refugee health professionals have gained in their home countries. De-skilling [32] and frustration [35] among refugee health professionals are often described in the context of labour market integration. Thus, it is necessary to make their resources and competencies visible again in order to increase their agency.

#### Improvement of formal skills

While there was strong agreement with improvement of language and professional skills, agreement with items in the category of formal resources was very mixed. The only item that was rated very high in this category referred to being encouraged to work again. Participants only partially agreed that they would find a job easier. This ambivalence may be explained by two factors. Several participants described that participation in the programme improved their CV which could help find a job. However, as most of the participants had not yet the achieved required language competencies and not participated in the mandatory language and knowledge assessment examination, it is not possible to predict whether participation in the programme will help finding a job.

Quantitative findings also reveal that most of the participants were able to build networks and qualitative findings indicate that participants stayed in contact with each other after three months. However, they did not report establishing networks with staff members or people already working in the health care system. Although participants in this programme benefitted from local students during the shadowing, they did not report staying in contact with them afterwards. Studies in the context of international students in Germany describe that establishing relationships with locals is perceived as challenging [36] and participants in this study described that they had a distanced relationship towards local team members. Although workplaces are described to be a good place to establish contacts [37], it remains unclear why no contacts were made. One explanation may be that due to language barriers the communication between local team members and refugee health professionals can be impeded. Another explanation may be the precarious staffing situation in health care. Participants in this programme were sometimes not even supervised appropriately which may be related to local team members being overburdened. Thus, the chances of establishing relationships between local team members and refugee health professionals may be low. However, another explanation for the distanced relationship is that local team members may be hesitant or not willing to establish contact with refugee health professionals. Negative attitudes towards refugees and asylum seekers are becoming stronger in Germany [38]. Thus, it is important to increase cross-cultural awareness within the organisation in order to create a welcoming environment that appreciates refugee health professionals [16].

Results also indicate that some participants did not experience a positive effect on self-confidence. Moreover, some of them were disappointed and discouraged through the programme. Based on qualitative findings, it can be concluded that those participants did not receive a shadowing placement, were not supervised appropriately or were not physicians. This matches the experience of the staff who had difficulty finding shadowing placements and proposed establishing mentoring programmes in order to ensure some kind of induction or attachment to the ward. Results indicate that in their practical year students were helpful tandem partner. It is recommended to implement mentoring or tandem programmes [14]. This could disburden employees at the ward but it could also assure some kind of attachment to the ward by matching them with students who are already working there. Additionally, refugee health professionals could be matched with former international or immigrated health professionals who have successfully integrated at their workplaces as role models can be an important resource at the workplace [39].

Most of the observed outcomes were also reported three months later. Although participants talked much more generically about the programme, a positive feeling remained with them. Nevertheless, the same applies to negative effects that were also reported three months later. Thus, it may be important to critically reflect on positive but especially on possible negative outcomes before designing and implementing programmes.

## Strengths and limitations

The strength of this study is the mixed methods study design that was implemented in order to capture the different facets of programme implementation and programme outcomes. By conducting quantitative and qualitative data collection, it was possible to extract detailed information on the improvement of the programme and the impact it had on participants. Through the mixed-methods design it was also possible to include all participants in the evaluation instead of selected participants. Furthermore, by conducting interviews at several times, recall bias was reduced. Moreover, by interviewing participants after three months, it was possible to determine long term effects. However, qualitative data can be influenced by social desirability bias. Moreover, the psychometric properties of the quantitative instruments have not yet been evaluated. Although the study cannot be generalised due to limitations, the study provides an important basis for researchers and practitioners who want to implement or evaluate a programme for refugee health professionals.

## Conclusion

This study presents the results of a process and outcome evaluation of an orientation programme for refugee health professionals. The division between job shadowing, technical language courses and cross-cultural coaching was generally perceived as helpful. Moreover, quantitative and qualitative results show that improvements were observed with regard to language skills, professional skills and network establishment. As some participants could not benefit from the programme, it is necessary to enable all participants a shadowing placement and facilitate supervised job shadowing placements. Moreover, programmes should focus stronger on reducing fear and anxiety in order to increase the agency of refugee health professionals and supporting their labour market integration.

## Notes

1. Participants were selected on the basis of their German language skills and the likelihood of completing the programme.

2. All interviews were conducted in person at the University Medical Center. The follow-up interviews were conducted via telephone as at this time point travel costs had been identified as a barrier for participants and we did not want the interview to be an additional burden.

3. The interviews were conducted in German. For translation purposes, citations were translated one-on-one from German to English and supplemented with further explanations in square brackets if necessary.

4. Although officially there were only 29 participants, we identified 30 codes for the German course. Despite a thorough examination, it was not possible to identify or match the double code. In order not to manipulate data, attendance rates for German course were based on 30 participants. Limitations of quantitative data are critically reflected in the discussion section.

5. In total, 251 out of 360 possible feedback from the 30 participants were available for evaluation. Thus, ratings in this context are dependent on participation and attendance rates.

6. There were four answer categories in the weekly questionnaires: (1) I am not satisfied with the course. (2) I am a bit satisfied with the course. (3) I am satisfied with the course. (4) I am very satisfied with the course. The value ‘mean’ in the following refers to the average values of the 12 weeks.

7. There are a total of 217 out of the possible 348 feedbacks (with a theoretical attendance of 100%). Thus, ratings in this context are also dependent on participation and attendance rates.

8. In the post questionnaire there were five answer categories: (1) Strongly disagree. (2) Disagree. (3) Partially agree. (4) Agree. (5) Strongly agree.
